# MicroDSC study of Staphylococcus epidermidis growth

**DOI:** 10.1186/1471-2180-10-322

**Published:** 2010-12-17

**Authors:** Dragos C Zaharia, Cezar Iancu, Alexandru T Steriade, Alexandru A Muntean, Octavian Balint, Vlad T Popa, Mircea I Popa, Miron A Bogdan

**Affiliations:** 1 University of Medicine and Pharmacy „Carol Davila", Eroii Sanitari Bulevard 8, Bucharest, Romania; 2 Romanian Academy, Institute of Physical Chemistry „Ilie Murgulescu", Splaiul Independentei 202, 060021, Bucharest, Romania

## Abstract

**Background:**

A microcalorimetric study was carried out using a *Staphylococcus epidermidis *population to determine the reproducibility of bacterial growth and the variability of the results within certain experimental parameters (temperature, bacterial concentration, sample thermal history). Reproducibility tests were performed as series of experiments within the same conditions using either freshly prepared populations or samples kept in cold storage. In both cases, the samples were obtained by serial dilution from a concentrated TSB bacterial inoculum incubated overnight.

**Results:**

The results show that experiments are fairly reproducible and that specimens can be preserved at low temperatures (1 - 2°C) at least 4 days. The thermal signal variations at different temperatures and initial bacterial concentrations obey a set of rules that we identified.

**Conclusion:**

Our study adds to the accumulating data and confirms available results of isothermal microcalorimetry applications in microbiology and can be used to standardize this method for either research or clinical setting.

## Background

Bacterial infections are a major public health problem [1-3]. Community acquired infections with multidrug resistant bacteria and especially hospital acquired infections, have a high mortality rate in the first days of hospitalization [4-6]. The mortality rate is increased by the use of inappropriate antibiotic therapy owing to the rather long duration for obtaining an antibiogram (between 24 and 48 hours).

Recent studies have proven the possibility of obtaining a quick (4-5 hours) and complete antibiogram by means of microcalorimetry [7-10]. The studies published so far are basically qualitative in nature, relying mainly on the presence or absence of microcalorimetric signal in media containing antibiotic. Other microcalorimetric studies have also described signal variation depending on bacterial concentration [11]. As emphasized in a recent review, microcalorimetry has the advantages of sensitivity and accuracy "for dynamic measurements of bacterial numbers that cannot be achieved with microscopic enumeration, plate counts or protein assays" [12].

The present contribution contains results obtained via differential scanning microcalorimetry (microDSC), a method related to isothermal microcalorimetry (IMC), utilized in recent studies in the form of high throughput, multi-channel (multi-sample) experimental setups [7-13]. Although microDSC is able to investigate only one sample on a single run, its versatility, expressed as heating-cooling and fluid mixing capabilities (within sensitivity performances similar to IMC), recommends this technique for research purposes. We have studied freshly prepared bacterial inocula as well as samples kept for 1 to 4 days at low temperature (1 to 2°C). In addition, our research aimed to study the variation of the calorimetric signal patterns with respect to working temperature and bacterial concentration.

Previous isothermal microcalorimetric studies indicate a time lag of approximately 1 hour between sample preparation and actual signal recording [9,12]. Within this time, reference and sample cell equilibration takes place. Minimizing the information loss regarding bacterial growth dynamics associated with this time lag is an additional objective of the present contribution. Finally, we have examined the sources of signal perturbation upon attempting to reduce the time lag and its influence on intrinsic bacterial thermal signal.

## Results

We studied the reproducibility and variability of the growth thermal signal of *Staphylococcus epidermidis*, as registered by Setaram microDSCIII.

In all figures, this thermal signal is expressed as Heatflow (mW) versus Time (h).

### The reproducibility of the Heatflow-Time behavior depends on the method used in sample preparation

**Figure 1:**
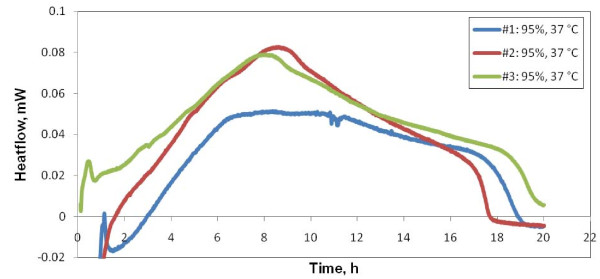
**Reproducibility test starting at room temperature (*fresh sample *experiments)**. Thermal signals of a series of successively freshly prepared samples of the same approximate transmittance (T600~95%). Reproducibility issues are generated mainly by sample preparation history. Instrument equilibration period was cut off the recording.

Our first attempts at studying the reproducibility of the signal were carried out using *freshly prepared samples*. These were directly introduced into the calorimeter which was allowed to equilibrate at 37°C. The growth thermograms of a series of samples of the same approximate transmittance are shown in Figure 1.

**Figure 2:**
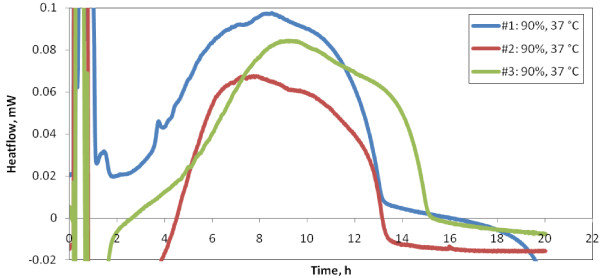
**Reproducibility test starting at low temperatures (*samples kept in cold storage*)**. Thermal signals of a series of samples of the same transmittance (T600 = 90%) kept in cold storage at 1 - 2°C. Reproducibility issues are mainly generated by the thermal regime, i.e. *iso - non-iso - iso *switches. Perturbations of the thermal signal are also evidenced in the figure. One may notice the partial overlap of these perturbations with the intrinsic thermal signal of the bacterial populations.

Regarding the reproducibility of the signal, the best results were obtained using *samples kept in cold storage *(described in **Methods**) as evidenced in Figure 2. This can be ascribed to the lack of thermal stability at the beginning of the experiments carried out with *freshly prepared samples*, as well as by errors encountered in sample preparation and transmittance measurements (pertaining to different inocula in the first case, while for *samples kept in cold storage *the same inoculum was used within a sample series).

The method used in sample preparation and sample *thermal history *play an essential role in signal reproducibility. The term *thermal history *refers to the duration of cold storage of the sample, calorimeter temperature at the moment of sample loading, the thermal program samples experience including cooling/heating rates utilized prior to the target isothermal regime.

### Variation of selected parameters leads to differences in the Heatflow-Time signal

A range of bacterial concentrations (as evidenced by T600) and working temperatures were used within the present study to assess the variability of the thermal signal generated by bacterial growth.

**Figure 3:**
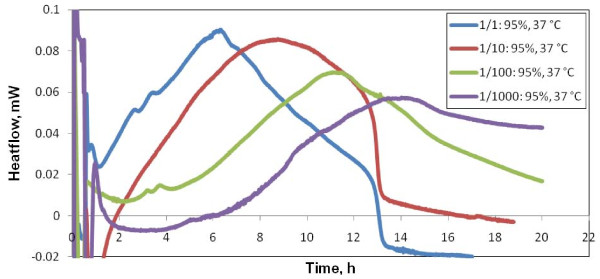
**Variability test starting at room temperature (*freshly prepared samples*)**. Thermal signals of serial dilutions, 1/10, 1/100, 1/1000, of samples of T600~95% incubated at a temperature of 37°C. Signals generated by bacterial populations of increasing dilution show decreasing signal height and longer time to signal appearance.

As evidenced in Figure 3 thermograms generated by bacterial populations of increasing dilution show decreasing signal heights and longer time lags associated with the appearance of sizable thermal effects. Although the starting concentration ("dilution = 1") is close to the transmittance detection limit (95%), even a further 1000-fold dilution of this initial sample generated measurable thermal signal. This confirms recently reviewed findings of the microcalorimetric high sensitivity, far beyond that of turbidity measurements [12]. The following growth pattern is observed: the time lag and extension of the thermal signal increase with increasing dilution. In the 1/1000 dilution case, sample growth is not completed within the chosen 20 hours experiment time limit.

**Figure 4:**
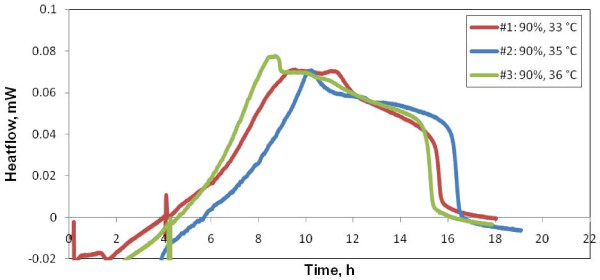
**Variability test starting at low temperature (*samples kept in cold storage *experiments)**. Thermal signal of a series of samples of the same transmittance (T600 = 90.1%) incubated at different temperatures: 33, 35 and 36°C. Thermal signal is obtained faster and is generally of higher intensity with increasing temperature.

Variability with temperature at a fixed transmittance is shown in Figure 4. Thermal signal is obtained faster, with slightly higher intensity with increasing of the growth (working) temperature. This follows the expected trend of growth rate increase with temperature.

### Sources of signal perturbation

The productive use of this method for the study of bacterial population dynamics entails the determination of the following important factors that might contribute to errors in generating data:

1. Sample preparation - we have encountered this error in *experiments on freshly prepared samples*. Storing the samples at low temperatures eliminates this error by using aliquots of the same bacterial preparation (as described in **Methods**).

**Figure 5:**
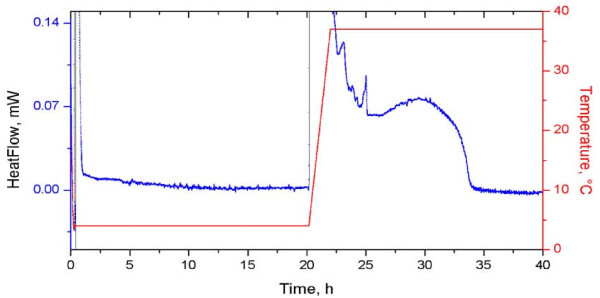
**Low temperature thermal inactivity check**. Thermal signal of a concentrated sample (T600 = 48%) submitted to the following thermal regime: (i) sample cell introduction at room temperature; (ii) cooling with 1 K/min to 4°C; (iii) 20 hours of isothermal maintaining at 4°C; (iv) ramp heating with 1 K/min to 37°C; (v) 20 hours of isothermal maintaining at 37°C. One can notice the thermal inactivity at 4°C followed by the "wake-up" of the bacterial population on heating. Perturbations caused by thermal switches are clearly overlapping with the intrinsic thermal signal of the bacterial population.

In this case one potential issue was the viability of the bacterial samples stored at low temperature for a considerable amount of time (up to four days). We designed an experiment to test the lack of bacterial metabolic activity at low temperatures (Figure 5). One may notice that there is no sizable thermal activity of the bacterial population isothermally kept at a 4°C for 20 hours. However, the bacterial population is viable, as evidenced by its thermal activity at 37°C.

Subsequent recordings using samples kept at low temperature for up to 4 days provided similar signals.

2. The response of the microcalorimeter to perturbations produced by sample loading. All experiments are affected by perturbations during sample loading that potentially can mask early stage bacterial growth. This is most evident in experiments on *freshly prepared samples *because of early detection of bacterial growth thermal signal. In Figure 1, the signal perturbation was cut off.

3. Perturbation during *iso - non-iso - iso *thermal switches. Due to ramp heating, experiments performed on *samples kept in cold storage *are mostly affected by switches in thermal program.

In Figure 5, the thermal "wake-up" of the bacterial population is masked by the inherent microDSC signal perturbation at *iso - non-iso - iso *thermal switches. This feature, also observed in Figure 2, can explain some of the reproducibility problems.

4. Rate of ramp heating. A slow heating rate favors early stage bacterial growth within the non-isothermal regime. In spite of this, signal perturbation at thermal switch is lower and is amenable to subsequent signal processing. Slow heating is particularly suitable for samples with low concentration, where early stages of bacterial growth are not thermally important. Higher rates of ramp heating produce larger perturbations at the thermal switch but lower overlap with signal generated by bacterial growth. These higher rates are suited for samples of higher concentrations, which generate a sizable early thermal signal. To optimize the time required for experiments and minimize overlap, a careful balance between these experimental parameters is necessary.

## Discussion

Microcalorimetry is quickly gaining recognition as a tool in microbiology. In this contribution we sought to investigate the reproducibility and variability of growth pattern measurements carried out on a reference strain of *Staphylococcus epidermidis*.

So far, many of the applications of microcalorimetry in medical science and research are qualitative in nature. Trampuz et al [11] have described a microcalorimetric method for the screening of platelet products for contamination. Daniels et al [13] point out that qualitative detection of bacterial growth is almost three times faster using microcalorimetry in a comparison with another commercially available rapid detection method. In both studies, positive diagnosis of bacterial growth was defined as a 10 μW increase in heatflow above baseline.

In our paper, we present the microDSC analysis of *Staphylococcus epidermidis *growth in TSB. Experiments on *freshly prepared samples *presented above mimic the above-mentioned isothermal microcalorimetric (IMC) experimental setups [7-13]. As we have shown, the overall reproducibility of the method was satisfactory for *fresh samples*; however, better reproducibility was obtained using *samples kept in cold storage*, mainly due to the lower thermal instability encountered at the start of signal recording. *Thermal history *appears to be an essential factor for the reproducibility of microDSC runs.

We have evidenced the variability of the growth thermal signal of *Staphylococcus epidermidis *with respect to initial concentration and isothermal growth temperature.

The time lag of growth detection and the overall time extension of the thermogram increase with initial sample dilution, whereas the heatflow amplitude decreases with the initial sample dilution (Figure 4). On the other hand, the time lag of growth detection and overall extension of the thermogram decrease with the working temperature, while the peak amplitude increase is less pronounced (Figure 5). This adds to observations of Trampuz et al [10], which showed, for cultures of *S. pneumoniae *and *L. monocytogenes*, that in instances where qualitative diagnosis of bacterial growth is necessary, adjustment of incubation temperature yields a faster result.

Microcalorimetry has real potential as a method for obtaining quick information about the antibiotic susceptibility of bacteria. In a recent publication, microcalorimetry was used to test the susceptibility of bacterial inocula to multiple antibiotics [9]. In a review paper Daniels at al [12] point out the advantages and drawbacks of microcalorimetry, its potential clinical use as well as research utility in environmental applications. This method is promising for clinical settings as shown by Baldoni et al [8] which tested the antibiotic susceptibility on clinical isolates of *Staphylococcus aureus*. Some essential factors affecting microDSC reproducibility as well as the advantages of this experimental technique were evidenced within this contribution. We consider that a detailed investigation (including kinetic analysis) of reproducible thermal signal of bacterial growth can lead to the development of alternative means of rapid bacterial identification and antibiotic susceptibility. Results of this ongoing study will be the object of subsequent contribution.

## Conclusions

The above results validate the microDSC technique as an alternative to the more productive multi-channel IMC. The method compensates its lower throughput with higher flexibility and ability to recognize sources of experimental errors and means to avoid them. Acceptable reproducibility on freshly prepared samples was obtained and the thermal perturbation generated by sample introduction at the working temperature was found as the main source of experimental errors for this method. Better reproducibility is achieved with samples of the same bacterial suspension (inoculum) preserved for up to 4 days in cold storage and introduced in the calorimeter at 4°C. The effects of bacterial suspension concentration and working temperature on growth thermal signal were identified. By means of a thorough thermal signal analysis the method can be developed and optimized for bacterial growth kinetics thus offering a potentially valuable tool for both research and clinical purposes.

## Methods

### Microcalorimetry

For our microcalorimetric studies we used a Setaram MicroDSC III differential scanning microcalorimeter, Joule effect factory calibrated. Outer thermostatic loop was provided by a Julabo F32-HE device operating in standard mode. 3D sensor protection was provided with Argon purge gas (99.99% SIAD - TP). Setsoft 2000 V 3.05 software was used for data acquisition and primary signal processing. In each experiment, a sample of 600 μL was introduced in a batch cell with a capacity of 1 mL (with a maximum sample volume of 850 μL).

### Bacterial population

We performed the microcalorimetric experiments on a strain of *Staphylococcus epidermidis ATCC 12228*.

### Culture medium

Bacterial cultures were prepared in trypticase soy broth (TSB) which is a mixture of Pancreatic digest of casein (17 g), NaCl (5 g), Papaic digest of soybean meal (3 g), K_2_HPO_4 _(2.5 g), Glucose (1.8 g) to 1 liter and a pH of 7.3 ± 0.2 at 25°C. The medium was autoclaved before use and microbiologically pure.

For bacterial plating, isolation and random sample checking of sterile conditions we used trypticase soy agar (TSA) which is a solid medium, with the same basic components as TSB.

### Sample preparation

Discrete colonies of *Staphylococcus epidermidis *grown on TSA culture media were used to prepare TSB cultures. For bacterial growth, the liquid suspensions were kept overnight at 37°C in the JulaboF32-HE thermostat. Subsequent inocula were prepared, with the desired transmittance measured at 600 nm *(T600)*. Depending on the experiment, serial dilutions of the inoculum were performed. The transmittance measurements were made using blank TSB as reference. TSA calibration of transmittance indicated a concentration of ≈5×10^7 ^CFU/mL for the T600 = 95% suspension, the most frequently used dilution within this study.

The sample cells and their hermetically o-ring sealing caps were sterilized at 121°C and kept sealed until use.

### Procedure

The sample cells were filled at room temperature and were hermetically silicon o-ring sealed. A batch cell containing 600 μL sterile TSB was used as reference for differential scanning microcalorimetry (μDSC).

Two types of experiments to test signal reproducibility and variability were performed:

#### a. Experiments on freshly prepared samples

Samples were prepared as described above and introduced in the microcalorimeter immediately after preparation. They were allowed to reach thermal equilibrium at room temperature. The working temperature was reached with maximum heating rate then kept constant for the entire experiment and the signal was recorded.

#### b. Experiments on samples kept in cold storage

A series of samples of the same transmittance, prepared as described above, were submitted to cold storage immediately after preparation And kept for 1 to 4 days at 1-2°C. The experiments were performed at 1 day intervals using these samples, while keeping the same reference cell. The microcalorimeter was allowed to reach thermal equilibrium at 4°C for about 15 min. The sample cells were then taken out of cold storage and rapidly introduced in the calorimeter; after additional 15 minutes for reaching thermal stability at 4°C the recording of the actual experiment started. Working temperature was reached by ramp heating at a rate of either 0.1 K/min or 1 K/min; isothermal runs' duration was typically 20 hours.

#### Low temperature thermal inactivity check

This experiment was devised to evaluate the thermal behavior of the bacterial population during manipulation/storage (Figure 5). A freshly prepared sample was introduced into the microcalorimeter, cooled down to 4°C, and then kept for 20 hours at this temperature. The temperature was then raised to 37°C by ramp heating with 1 K/min, and kept at this temperature for another 20 hours.

## Abbreviations

TSA: Tryptic Soy Agar; TSB: Tryptic Soy Broth; T600: transmittance measured at 600 nm.

## Authors' contributions

DCZ carried out bacterial cultures and inocula preparation, data processing and analysis. CI carried out microDSC experiments and data processing. ATS carried out microDSC experiments and data processing. AAM carried out bacterial cultures and inocula preparation, microDSC experiments and data processing and analysis. OB carried out bacterial cultures and inocula preparation, microDSC experiments and data processing and analysis. VTP initiated and conceived this study, designed and supervised microDSC experiments and data analysis. MIP initiated and conceived this study, designed and supervised bacterial growth. MAB initiated and conceived this study, supervised the preparation of the manuscript. All authors participated in drafting of the manuscript and approved its final form.
